# Long-term effect of a GnRH-based immunocontraceptive on feral cattle in Hong Kong

**DOI:** 10.1371/journal.pone.0272604

**Published:** 2022-08-17

**Authors:** Rebecca Pinkham, Ka-Kei Koon, Jason To, Jason Chan, Flavie Vial, Matt Gomm, Douglas C. Eckery, Giovanna Massei

**Affiliations:** 1 National Wildlife Management Centre, Animal and Plant Health Agency, York, United Kingdom; 2 Agriculture, Fisheries and Conservation Department, Animal Management (Operation) Division, Hong Kong SAR, China; 3 USDA APHIS, National Wildlife Research Center, Fort Collins, Colorado, United States of America; Universite Clermont Auvergne, FRANCE

## Abstract

Increasing human-wildlife conflicts worldwide are driving the need for multiple solutions to reducing “problem” wildlife and their impacts. Fertility control is advocated as a non-lethal tool to manage free-living wildlife and in particular to control iconic species. Injectable immunocontraceptives, such as GonaCon, stimulate the immune system to produce antibodies against the gonadotrophin-releasing hormone (GnRH), which in turn affects the release of reproductive hormones in mammals. Feral cattle (*Bos indicus* or *Bos taurus*) in Hong Kong are an iconic species whose numbers and impacts on human activities have increased over the last decade. Previous studies have proven that a primer vaccination and booster dose of GonaCon in female cattle are safe and effective in reducing pregnancy levels one year post-treatment. The aims of this project were 1. to evaluate the longevity of the effect of GonaCon in feral cattle up to four years post-vaccination; and 2. to assess if a second booster dose of GonaCon, administered at either two or four years post-vaccination, extends the contraceptive effect in this species. Vaccination with GonaCon, administered as a primer and booster dose, was effective in causing significant infertility in free-living cattle for at least three years post-vaccination, with the percentage of pregnant animals in the vaccinated group decreasing from 76% at vaccination to 35%, 19% and 7% in years 2, 3 and 4 post-vaccination, compared with 67% at vaccination to 50%, 57% and 14% respectively in the control group. A second booster dose of GonaCon administered either 2 or 4 years after vaccination rendered 100% of the Treated cattle infertile for at least another year. These results suggested that vaccination with GonaCon can reduce feral cattle population growth and that a second booster dose can extend the longevity of the contraceptive effect.

## Introduction

Human-wildlife conflicts are increasing worldwide and are often associated with overabundant animal populations [1–4]. Traditional methods used to mitigate these conflicts include culling and toxicants. However, public opposition to lethal methods is growing, mainly driven by animal welfare concerns, human safety in urban settings and environmental impact of some of this methods [5–7]. This has fostered interest in alternative options, such as fertility control, to manage overabundant animal populations and in particular iconic species [8–11]. In this context, injectable immunocontraceptive vaccines are increasingly advocated as alternative or complementary to culling for managing wildlife [12–14]. These vaccines act by inducing antibodies to proteins or hormones essential for reproduction.

One of these contraceptives, an injectable, single-dose gonadotropin releasing hormone (GnRH) vaccine, GonaCon, has been consistently shown to decreased fertility for one to six years in several ungulates including white-tailed deer (*Odocoileus virginianus*), wild boar (*Sus scrofa*), horses (*Equus caballus*), and bison (*Bison bison*) [10, 15–19]. GonaCon induces an immune response to the GnRH, which in turn affects the cascade of reproductive hormones that lead to ovulation: following vaccination with GonaCon, females do not exhibit oestrous [16]. In most species, the contraceptive effect of the vaccine decreases with time [18, 20, 21] although infertility can be maintained for multiple years (e.g. [18, 22, 23]).

The number of free-roaming feral cattle (*Bos indicus or Bos taurus*) in Hong Kong increased in the last decades, in parallel with their impact on human activities. Cattle were traditionally used as draught animals, but in the 1950s, due to a decline in agricultural activities, these animals were released into the wild by local farmers. At present, most free-roaming feral cattle are found away from the highly urbanised areas of Hong Kong. The impacts of feral cattle include traffic disturbance and accidents, environmental nuisance and crop damage. However, cattle are valued as local heritage and some Hong Kong stakeholders support non-lethal control to manage these populations.

In 2011, the Agriculture Fisheries and Conservation Department (AFCD) set up a Cattle Management Team for the long-term management of feral cattle, which in 2013 were estimated to be circa 1100 individuals. The AFCD explored surgical sterilisation that was initially achieved by capturing and transporting feral cattle to the Cattle Team Operation Center, where animals were surgically sterilised, and either returned to their natal area or relocated to country parks. As capture and transport of cattle are impossible in remote areas, a programme of immunocontraception started in 2013.

The initial studies, carried out with captive and free-living cattle, showed that vaccination with a primer and a booster dose of the injectable immunocontraceptive GonaCon did not cause any observable adverse reactions [24, 25]. In particular, the vaccine did not appear to interrupt ongoing pregnancies but reduced fertility significantly, as the proportion of pregnant cattle in the GonaCon-treated group decreased from 76% at initial vaccination to 6% one year after vaccination, compared to 67% and 57% respectively in the control group.

These studies confirmed that GonaCon is safe and effective in inducing infertility in feral cattle and that a booster dose, administered 3–6 months after the first vaccination, was critical for maintaining infertility. The next phase of the study tested the long term effectiveness of a vaccination/booster combination with GonaCon across multiple years to assess the long-term potential impact of GonaCon treatment in cattle populations.

The objectives of this study were: 1. to evaluate the longevity of the effect of the immunocontraceptive treatment in feral cattle up to four years post-vaccination; and 2. to assess if a second booster dose of GonaCon, administered at either two or four years post-vaccination, extends the contraceptive effect in this species.

## Methods

The study monitored reproduction in two cohorts of female feral cattle, previously assigned to Treatment and Control groups. The first Cohort was included in the study in summer 2015, with 42 animals Treated (T) with GonaCon and 18 used as Control (C) as described in Massei, Koon [24]. Treatment consisted of an intramuscular injection of 3 ml of GonaCon (1000ug/ml of GnRH-mollusk-hemocyanin conjugate; lot number: GCRD05262015; USDA, Fort Collins, CO, USA) in the neck. Control animals were injected with an equivalent volume of a 0.9% saline solution (Sodium Chloride Injection; Thai Otsuka Pharmaceutical Co. Ltd., Thailand). A booster dose of 1 ml of GonaCon was given 3–6 months later to all recaptured Treated cattle, and Control group were injected with 1 ml of saline solution.

A second Cohort of 25 Control and Treated animals was added in 2017 and boosted within 12 months. Treated animals in the second Cohort were considered control/untreated animals at first capture for comparison with Cohort 1.

To monitor the long-term effects of GonaCon, the Cattle Team attempted to locate and sample study animals once per year in summer 2016, 2017, 2018, 2019 and 2020 (Table 1). Cattle were anaesthetized and a sample of 24 ml of blood collected from each animal.

**Table 1:** Schedule of sampling and number of female feral cattle sampled at each stage in Cohorts 1 and 2.

	Cohort 1		Cohort 2	
	Treated	Control	Treated	Control
2015 Summer	42	18	-	-
2015 Winter	38	13	-	-
2016	33	14	-	-
2017	26	9	17	8
2018	16	7	15	7
2019	14	3	13	4
2020	11	7	9	5

The effectiveness of the vaccine to induce long-term infertility was monitored using the immune response to the vaccine, assessed by measuring serum anti-GnRH antibodies, and by pregnancy status. *Ad-hoc* observations by the Cattle Team or by local Non-Governmental Organizations (NGOs) on the reproductive output of study animals were also added to the database.

Serum samples were used to quantify the anti-GnRH antibody titres and to assess the reproductive status of treated and control animals. A pregnancy test, based on Pregnancy-Associated-Glycoprotein assay (PAG), routinely employed with farm cattle, was used [26]. The Rapid Visual Pregnancy Test kit (IDEXX Laboratories, Inc., USA) detects PAGs in bovine serum, which are expressed from the second month of gestation until calving. The limitations of this test are that PAGs can be detected 28 days post-breeding at the earliest, thus early pregnancy cannot be determined, and PAGs have a half-life of 60 days, therefore a positive result may occur up to 60 days post-calving. Therefore, the pregnancy status of cattle was interpreted based on both the PAGs pregnancy test and on direct observations of births of calves in the field. The estimated age of any calf at foot, based on body size, was also used to interpret PAG test results for female that may have given birth within 60 days of the pregnancy test.

An enzyme-linked immunosorbent assay (ELISA) (described in Massei, Koon [25]), was used to measure anti-GnRH antibody titres. Anti-GnRH antibody titres were expressed as the highest dilution at which the post-vaccination sample had a higher absorbance value than the mean (+2 SD) value of the pre-vaccination samples. The technician performing the ELISA tests was blinded to original treatment group of each serum sample.

### Statistical analyses

The proportion of pregnant cattle in Treated and Control groups in Cohort 1 was compared using one-sided Fisher’s exact tests. For treated animals that were sampled at least twice post-vaccination, the proportion pregnant was compared at vaccination and in subsequent sampling sessions using the McNemar’s exact test.

To establish a titre threshold for infertility, a ROC (receiver operating characteristic) curve was built that illustrates the ability to correctly identify individuals as pregnant based on antibody titres across Treated cattle in Cohort 1 and Cohort 2. There are several criteria for determination of the most appropriate threshold value based on ROC curves [27]. For this study, a titre threshold with a sensitivity >95% (i.e. with a low likelihood of false negatives) was chosen.

Using data from both Cohorts, the effects of age, season, and treatment on the probability of an individual cattle being pregnant was assessed through the fitting of univariable generalised linear mixed models with “Pregnant (Yes/No)” as the response variable and the animal ID as a random effect.

The longevity of the contraceptive effect of GonaCon was defined as the time it takes for a vaccinated animal to become pregnant again in cattle which had been sampled at least 3 times throughout the study. The time to the first pregnancy, excluding pregnancy at the time of vaccination, was computed for both Treated and Control animals. Survival curves for the time to become pregnant were fitted using the *survival* package [28] in R [29] and the mean survival time (= time to event in days) was derived from those curves. Differences in survival times between Treated and Control groups were analysed by fitting a Cox-proportional hazard model.

The tests described above were carried out in SPSS (version 25, IBM Corp, 2017) and R (version 3.6.1).

The study was approved in the UK by the Food and Environment Research Agency Ethical Review Process (ERP, 15/11/2012). As the AFCD in Hong Kong is a government department and not primarily a research institution, it does not have a permanent named IACUC: for this reason, an ad hoc Ethic Review Panel was set up specifically for this trial.

## Results

### Immune response to vaccination

In Cohort 1, 100% of the animals treated with GonaCon in 2015 had maintained anti-GnRH antibody titres two years post-vaccination (Table 2). Only three individual cattle had titres which fell below detectable levels by the fourth year post-vaccination, whilst all other recaptured cattle (n = 11) maintained detectable antibody titres.

**Table 2:** Number of treated cattle in Cohort 1 and relative anti-GnRH antibody titres at each time point following initial vaccination.

	Anti-GnRH antibody titre level (1:X,000)										N	% with titre
	NT	2	4	8	16	32	64	128	256	256+		
Vaccination	42										42	0
2 years post-vaccination			1	1	8	9	3	4			26	100
3 years post-vaccination	1			3	7	3	2				16	93.75
4 years post-vaccination	3	1	1	2	3	1		1	2		14	78.57

Animals were treated with a primer vaccination and a booster dose of GonaCon in 2015. NT = no detectable titre; N = sample size.

### Long-term effect of treatment on fertility

**Fig 1:**
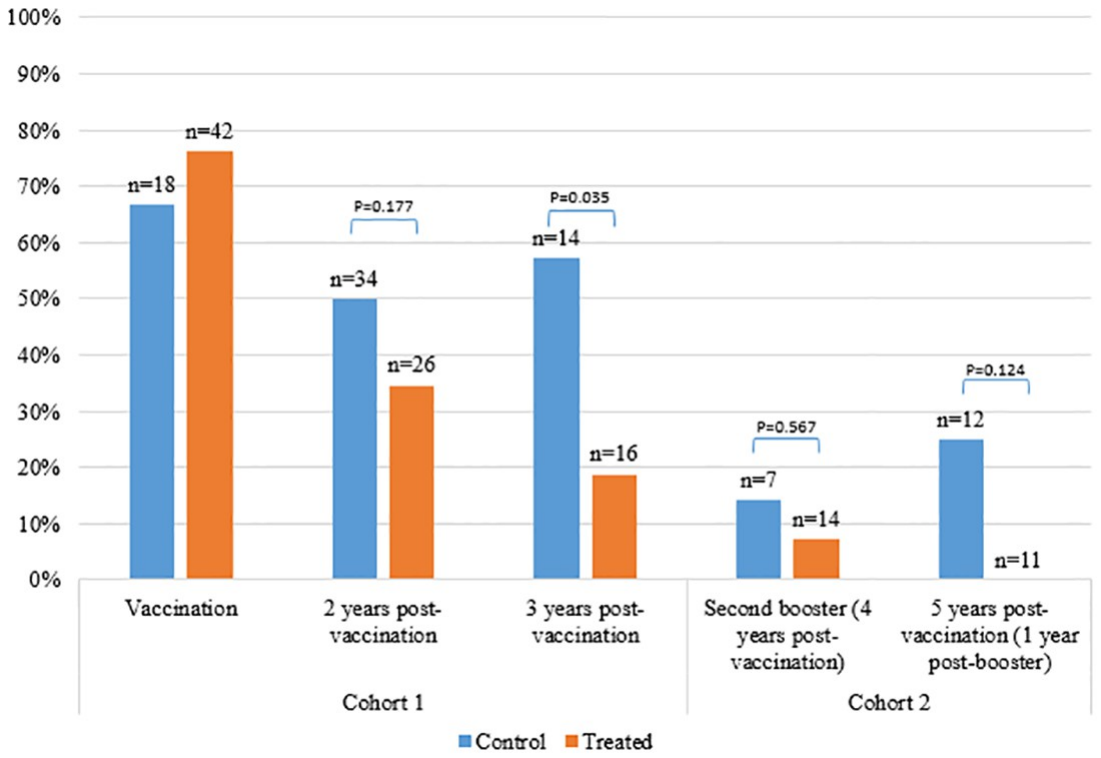
Percentage of treated cattle in Cohort 1 recorded as pregnant at sample points over time following vaccination with GonaCon in 2015, compared to untreated (control) cattle. Control group includes all untreated females captured at sampling point in Cohort 1, and additional untreated cattle, added from Cohort 2, included from 2 years post-vaccination. Treated cattle were administered a booster dose 3–6 months and 4 years after initial vaccination. Group sample size indicated on each bar.

The number of pregnant cattle was compared between Cohort 1 Treated animals and all untreated animals in the years following primer vaccination and booster dose in cattle. The percentage of pregnant animals in the Treated group was 35% in Year 2, 19% in Year 3 and 7% in Year 4, compared to 50%, 57%, and 14% in Control cattle respectively (Fig 1). There was a significantly lower proportion of cattle pregnant in the Cohort 1 Treated group than in Control animals in Year 3 post-vaccination (Fisher’s exact test, P = 0.035), although not in Year 2 (Fisher’s exact test, P = 0.177).

There was a significant effect of treatment with GonaCon on the likelihood of cattle being pregnant (univariate GLMM, O.R. 0.39 (0.21–0.74), P = 0.004), with Treated cattle less likely to be found pregnant than Controls at all recaptures post-vaccination across both cohorts.

For Treated cattle in Cohort 1, sampled at least once post-vaccination, the proportion of animals found pregnant at vaccination was significantly higher than the proportion observed pregnant in all subsequent sampling sessions (Exact McNemar’s: Year 2, P = 0.035; Year 3, P = 0.004; Year 4, P = 0.002; Year 5, P = 0.004). In comparison, for Control cattle that were sampled at least twice across the same time period, the proportion of animals found pregnant was not statistically different between vaccination stage and all subsequent sampling sessions (Exact McNemar’s: P>0.05 all years).

There was no effect of season (wet: May-Oct; dry: Nov-April) on the likelihood of a cattle being pregnant (P = 0.21). There was a significant effect of individual age on the likelihood of pregnancy (P = 0.05), with the odds of a pregnancy falling by nine percent per year of age. However, individual age at time of primer vaccination had no significant effect on the scale of immune response to the vaccine over time, indicating that vaccination is no less effective depending on age at start of treatment (P = 0.33).

### Pregnancy in relation to titre level

**Fig 2:**
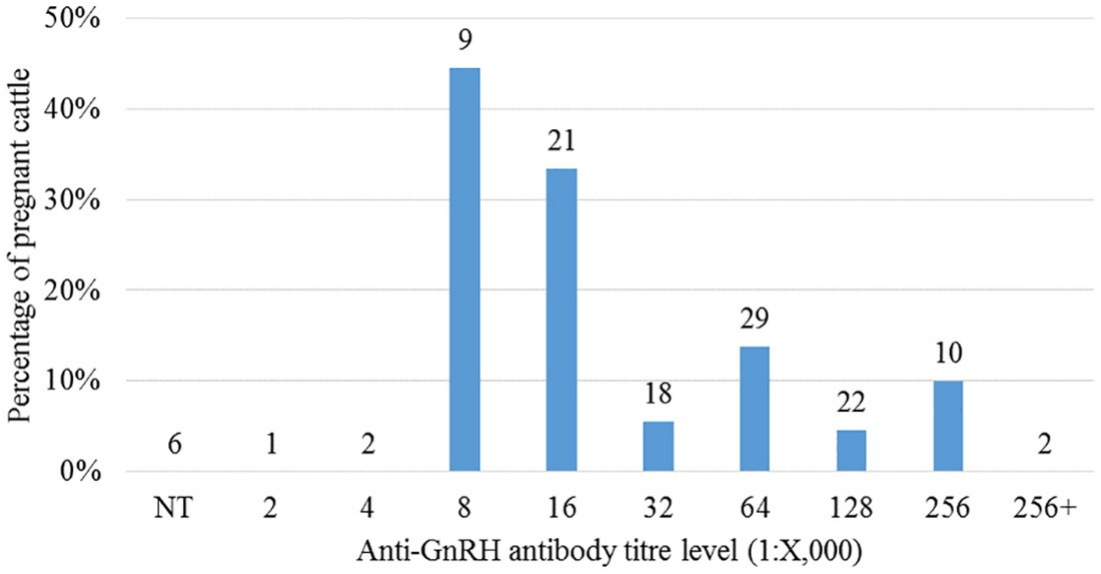
Percentage of treated cattle pregnant in relation to anti-GnRH antibody titres across all years post-booster. N = total number of samples at each titre level. NT = no detectable titre. Group sample size indicated on each bar.

In all Treated cattle sampled across all years, there was a negative association between the percentage of cattle recorded as pregnant and the levels of anti-GnRH antibody titres (Fig 2). Although there were no pregnancies recorded for the lowest titre levels (no detectable titre, 2K, and 4K) it is important to note that these titres were recorded in animals that in previous years had exhibited relatively higher titre levels between 32K and 256K.

**Fig 3:**
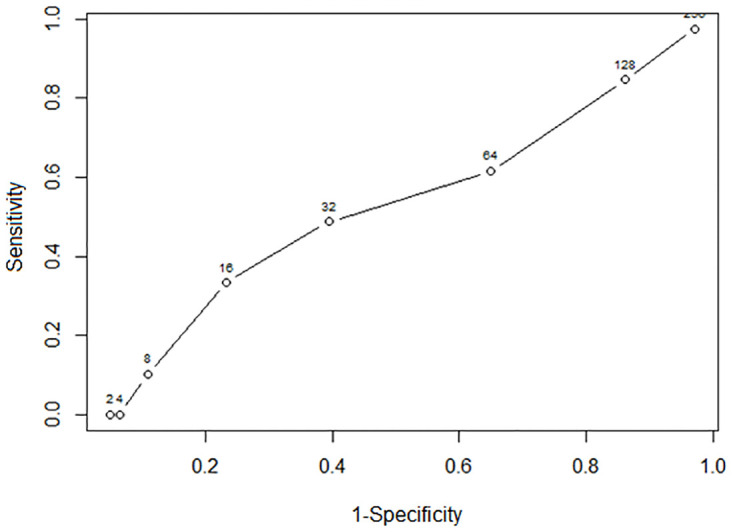
Receiver operating characteristic (ROC) curve for predicting infertility in relation to serum anti-GnRH antibody titres (1:X,000) in cattle.

The ROC curve obtained to identify an antibody titre threshold for infertility in cattle (Fig 3), indicated that if the criterion of sensitivity >95% was applied, a titre threshold >128K is required, the trade-off being very low specificity (3%), i.e. a large number of false positives (non-pregnant individuals with titres below threshold). In practice, this means that GonaCon-treated cattle with anti-GnRH titres above 128K are likely to be infertile, but if the titres are lower than 128K, animals might still be infertile or have become fertile.

### Effect of second booster dose

**Fig 4:**
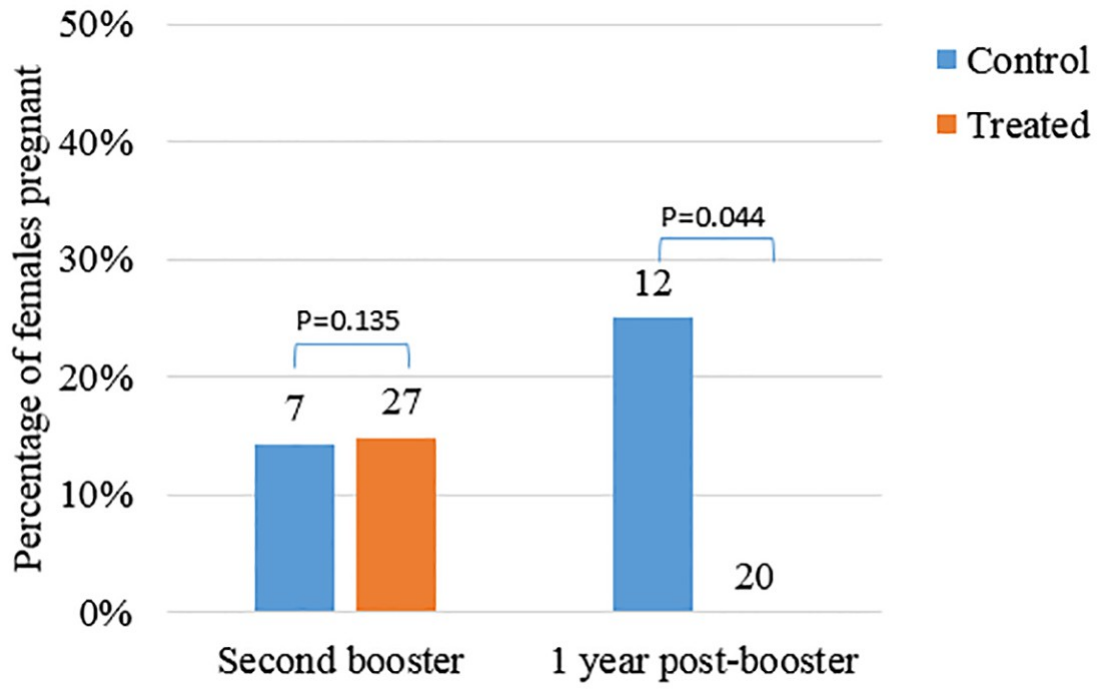
Percentage of pregnant treated and control cattle sampled in 2019 and in 2020. In 2019 cattle were administered a second booster dose of 1 ml of GonaCon, either two or four years after primer vaccination. N = total group sample size.

Following a second booster dose of 1 ml of GonaCon in 2019 to Treated animals in Cohort 1 and Cohort 2, the percentage of Treated cattle recorded as pregnant fell from 15% of 27, to 0% of 20 recaptured one year later (2020). Conversely, the percentage of all Control cattle recorded as pregnant in the same period rose from 14% to 25% (Fig 4).

Across both cohorts all females recaptured one year after a second booster dose of GonaCon displayed antibody titres (Table 3). A Wilcoxon signed rank test revealed a difference in titre level, with titres one year after the second booster significantly higher than those measured at the time of boosting (V = -1.5, P<0.001).

**Table 3:** Number of animals expressing anti-GnRH antibody titres in GonaCon Treated cattle, measured in samples collected one year after animals were administered a second booster dose of Gonacon in 2019.

	Anti-GnRH antibody titre level (1:X,000)										
	NT	2	4	8	16	32	64	128	256	256+	N
At second booster	1	1	1	2	5	1	5				16
1 year post-booster						1	8	4	3		16

Combined cohorts of cattle originally treated and boosted in 2015 (Cohort 1) or treated in 2017 and boosted in 2018 (Cohort 2). NT = no detectable titre; N = total sample size.

## Discussion

This study demonstrated that two doses of GonaCon (primer and booster, the latter administered within one year after primer), are safe and effective in causing significant levels of infertility in free-living cattle for at least three years post-vaccination. The lack of significant difference in the proportion of pregnant cattle in Treated and Control groups in Year 4 (2019) might be due to several factors discussed below.

A fertility threshold of 128k anti-GnRH antibody titres was identified, above which it is highly likely that the majority of cattle are infertile. Across all years, only one female was recorded as pregnant with a titre over 128K. However, titres equal or below the 128K threshold do not necessarily indicate that a cow has become fertile again. The fertility threshold of levels of anti-GnRH antibody titres >128k, identified for cattle in this study, could be used as an indicator of the long-term contraceptive effect of GonaCon. A fertility threshold of >64k in anti-GnRH antibody titres was observed in GonaCon-treated white-tailed deer [15], whilst this threshold was found to be >256k in GonaCon-treated laboratory rats [30]. Similarly, a strong negative association between anti-GnRH antibody titres and fertility was found in female elk and in laboratory rats treated with GonaCon [30, 31]. These thresholds should be interpreted with caution, even within a single species, as infertility at a high titre is highly likely but not guaranteed; conversely, animals with titres lower than the species-specific threshold may still be infertile.

A single dose of GonaCon induced multi-year infertility in numerous ungulate species such as free-ranging elk (*Cervus elaphus*) [21, 31], bison (*Bison bison*) [19], white-tailed deer (*Odocoileus virginianus*) [20, 32], wild boar (*Sus scrofa*) [17, 18] and feral horses (*Equus caballus*) [10, 16]. Although the longevity of effect appears to be species-specific, in all instances booster vaccinations resulted in higher levels and longer-lasting antibody production due to the anamnestic (cell memory) response [33]. The results of the present study indicated that a second booster dose administered 2–4 years after vaccination rendered all animals infertile for at least another year. Similar results were found in free-ranging horses immunised with a single dose of GonaCon in 2009 and re-immunised four years later [34]. After the first dose of GonaCon, the proportion of treated mares that foaled was lower than that of control mares up to the third post-treatment foaling seasons but four years after vaccination this proportion was similar in treated and control mares. Following a booster dose administered 4 years after the primer vaccination, the proportion of mares giving birth ranged from 0% to 16% for three consecutive years. The authors concluded that practical application of this vaccine in feral horses would require an initial inoculation to suppress fertility in a proportion of the mares, followed by re-immunization that would result in greater reduction in population growth rates. Future research in Hong Kong should monitor the long-term contraceptive effects of GonaCon on cattle administered a second booster, as it is possible the effects of the second dose will last a few years.

In species for which fertility control is required to reduce or halt population growth, the gradual reversibility of the contraceptive effect, at least in a proportion of animals can be regarded as desirable [12]. For instance, Druce, Mackey [13] employed immunocontraceptives to increase the inter-calving interval in female elephants. By rotating the animals vaccinated with the contraceptive, managers ensured that all females had the opportunity to reproduce, albeit at a slower rate, and thus could contribute their genes to the elephant population. As GonaCon-vaccinated cattle in Hong Kong can be identified by their ear tags, the same concept could be applied in this context, allowing GonaCon-Treated cattle that have returned to fertility to give birth whilst treating new animals. As the age of pregnant cattle in this study varied between 10 months and 13 year old, maximum calving age would need to be considered when rotating treated animals.

In recent years a new surgical sterilization programme has been implemented in Hong Kong that can be carried out *in situ* and thus it does not require feral cattle to be transported to a different location. Although this method has a key advantage over immunocontraception, namely leading to permanent sterilization, it also effectively removes the genetic contribution of an animal. Depending on the proportion of animals surgically sterilized, this method might reduce the genetic variation of this population. For instance, a study carried out on Japanese Polled feral cattle, which are maintained in small populations, found relatively low genetic diversity in these breeds and attributed this to the decreasing population size in the last three decades [35]. Hong Kong cattle show heterogeneous morphology, both in body type, size and coat colour. A recent analysis of the genotype showed that Hong Kong feral cattle have relatively high levels of genetic distinctiveness and stressed the conservation value of this population [36]. In this context, GonaCon could be used to increase time between births, with most cattle contributing to the population’s gene pool. To reduce the monetary and welfare cost of anaesthetising cattle to administer booster doses, darts with radio-transmitters could be employed.

At the start of the study, based on anecdotal field observations, it was assumed that at least 70% of the cattle would be pregnant each year. This agreed with a study on the percentage of pregnant cattle on Amsterdam Island (Indian Ocean) reporting that 73% of free-roaming cattle were pregnant per year [37]. Data collected in Hong Kong between 2015 and 2018 indicated that the percentage of control cattle pregnant each year varied between 73% and 50%. The decline of the percentage of Control cattle pregnant 2019 (14%, n = 7) and in 2020 (25%, n = 12) was unexpected. This decline might be due to 1. environmental factors, such as temperature, precipitation or availability of food, affecting reproduction of cattle for two consecutive years; 2. natural variation in the proportion of pregnant cattle; and 3. potential effects of surgical sterilisation on the bulls.

No obvious differences were apparent in environmental factors such as the mean annual temperature, mean rainfall and number of heavy rainy days in Hong Kong (http://www.hko.gov.hk/en/climate_change/climate_change_hk.htm) between 2014–2018 and 2019–2020. It is also improbable that the reproductive output was due to natural inter-year variation as the first four years of study the proportion of cattle reproducing had been relatively consistent. In the 2010–2020 decade 301 males and 402 females underwent surgical sterilisation. Based on the current total cattle population in Hong Kong, estimated by the Cattle Team to be 905 cattle, with a 1:1 sex ratio and assuming circa 77% of the bulls sterilised were still alive in 2019 and 2020 (Cattle Team, pers. comm.), approximately 62% of the bulls in the Hong Kong population are currently sterilised. Anecdotal evidence from field observations suggest that castrated bulls do not attempt mating but they will fight other bulls approaching the females. This reflects the behaviour observed in castrated males of other species. For instance Price, Adams [38] found that castration significantly decreased but did not eliminate the aggressive behaviour of bulls neutered at a pre-pubertal age and Hart and Jones [39] found that castrated male goats showed a long-term retention of sexual activity after castration. It is conceivable that the decline in number of pregnant cattle in Hong Kong might be due to sterilised bulls preventing other males from mating. Further field data should be collected to test this hypothesis. If true, this would be an important factor in assessing the effects of sterilisation of bulls on population dynamics. However, although the presence of sterilized males may have contributed to a reduction in overall pregnancies, the inference into the effect of GonaCon in these herds remains valid. The presence of a proportion of males across the area that remained intact (approximately 38%) should have resulted in pregnancies in Treated females in the final year at a comparable rate to the Control females, whereas this study found a significant difference, with 0% of Treated females calving following the second booster dose compared to 25% of the Control females.

As the management of feral cattle in Hong Kong is carried out to achieve a reduction in numbers but not remove these animals, choosing the best population control option will depend on the target population size and on the time this should be achieved. This study suggested that a combination of on-site surgical sterilisation and vaccination with Gonacon, as indeed carried out at present, might be the best option to manage feral cattle in Hong Kong and promote coexistence between human activities and this iconic species.

## Supporting information

S1 Dataset (XLSX)
